# Unsafe Breast Augmentation Practices and the Surge of Breast Infections - An Emerging Public Health Concern in Pakistan

**DOI:** 10.12669/pjms.42.2.14628

**Published:** 2026-02

**Authors:** Safna Naozer Virji, Lubna Vohra

**Affiliations:** 1 Dr. Safna Naozer Virji, FCPS General Surgery. Fellow, Breast Surgery, Department of Surgery, Aga Khan University Hospital, Karachi, Pakistan; 2 Dr. Lubna Vohra, FCPS, MRCS, FACS. Associate Professor, Section of Breast Surgery, Department of Surgery, Aga Khan University Hospital, Karachi, Pakistan

We are all familiar with how breast cancer is a major concern and on the rise in Pakistan, with an increasing number of women being diagnosed at a younger age than that seen in neighbouring countries. And while breast conservation, oncoplastic and breast reconstruction surgery are now routinely offered to breast cancer patients for improved aesthetic and psycho-social outcomes, there is a parallel, rising trend and desire among healthy Pakistani women for breast augmentation for a more attractive and youthful appearance.

As a relatively conservative and patriarchal society, customarily, women in Pakistan were restricted from getting personal aesthetic procedures. Nevertheless, with education, awareness and women’s empowerment, there has been a gradual shift in attitude, with women taking charge of their well-being and a rise in behaviour favouring personal care and an emphasis on aesthetics.

Traditionally, in Pakistan, breast augmentation has been an elective surgical procedure performed by a plastic surgeon under general anaesthesia and involved the use of implants and/or fat grafting. One of the only studies reported from Pakistan in 2017 describes these procedures and some of the adverse outcomes associated with them, like infection (6.4%), capsular contracture (2.3%) and implant removal (2.3%).^1^ Scars are another big concern when it comes to aesthetic surgeries, and various techniques for implant placement have been employed, like the trans-axillary, infra-mammary and even trans-umbilical approaches, to minimise the evidence of any breast augmentation.

The use of breast fillers for augmentation, though introduced as early as the 1950s in the West, was a novel phenomenon in our country until recent years. With the introduction of aesthetic and cosmetic clinics, breast augmentation with fillers has now become a trend among women (especially young women). The appeal of the procedure is that it is a minimally invasive procedure (no scarring), performed under local anaesthesia with a quick recovery time. Furthermore, it is noted to have a more natural augmentation as compared to implants, and most importantly, it is a more economical option. Where breast implant surgeries may cost Rs. 300,000 - 500,000/-, certain clinics offer breast augmentation with fillers for as low as Rs. 50,000/-.

The main types of breast fillers used in Pakistan are hyaluronic acid fillers and Bio-Gel fillers (e.g., polyacrylamide hydrogel (PAAG)), with hyaluronic acid fillers being the most commonly used substance. These substances are to be injected in the sub-glandular plane or the sub-pectoral plane. Though an attractive and economical option, it is not without complications. These include induration and palpable masses or lumps noted in up to 83.6% of cases.^2^ The incidence of infection and inflammation has been reported as high as 28% in literature which may progress to breast abscesses or even sepsis, often necessitating prolonged treatment or surgical intervention. This frequently results in breast distortion and scarring, compounding the cosmetic and psychological burden on affected individuals.^2^,^3^

Infective complications after breast fillers, requiring incision and drainage and debridement, are a rising concern faced by breast surgeons in Pakistan. Furthermore, the inadequate injection of the filler in the breast gland (parenchyma) instead of the sub-glandular plane leads to inflammatory responses and complications in the breast, necessitating surgical intervention with its associated morbidity.

This is a serious concern, as more and more women are electing to undergo these breast augmentation procedures, and with no national regulatory body to maintain standards of the procedures performed. There is a rising rate of complications seen among these women with significant and morbid results due to the surgical management of these adverse events. There is an urgent need for regulation, public awareness, and safer cosmetic practices to curb these complications associated with breast augmentation using breast fillers.

## Authors’ Contribution:

**SV** is responsible for the manuscript writing. **LV** is responsible for the concept, review and final approval of manuscript.
